# A Comparison between Ultraviolet Disinfection and Copper Alginate Beads within a Vortex Bioreactor for the Deactivation of Bacteria in Simulated Waste Streams with High Levels of Colour, Humic Acid and Suspended Solids

**DOI:** 10.1371/journal.pone.0115688

**Published:** 2014-12-26

**Authors:** Simon F. Thomas, Paul Rooks, Fabian Rudin, Sov Atkinson, Paul Goddard, Rachel M. Bransgrove, Paul T. Mason, Michael J. Allen

**Affiliations:** 1 PML Applications, Prospect Place, The Hoe, Plymouth, United Kingdom; 2 Plymouth Marine Laboratory, Prospect Place, The Hoe, Plymouth, United Kingdom; 3 Protein Technologies Ltd, Williams House, Lloyd St North, Manchester, United Kingdom; NERC Centre for Ecology & Hydrology, United Kingdom

## Abstract

We show in this study that the combination of a swirl flow reactor and an antimicrobial agent (in this case copper alginate beads) is a promising technique for the remediation of contaminated water in waste streams recalcitrant to UV-C treatment. This is demonstrated by comparing the viability of both common and UV-C resistant organisms in operating conditions where UV-C proves ineffective - notably high levels of solids and compounds which deflect UV-C. The swirl flow reactor is easy to construct from commonly available plumbing parts and may prove a versatile and powerful tool in waste water treatment in developing countries.

## Introduction

Ultraviolet (UV) disinfection is an established and effective means of disinfecting microbe-contaminated waste streams, and thus is widely utilised in sewerage treatment [1], [2], cooling water tower disinfection [3], treatment of potable water in disaster affected areas [4], and even wound sterilisation [5]. Despite the undoubted effectiveness of UV sterilisation in these areas, it’s widespread application is limited by environmental factors which impair the transmission of UV light, such as colour [6], presence of humic acids [7], [8] and suspended particles [9]–[11]. Evidence indicates such factors can lead to sub-lethal exposure of microbes followed by their subsequent reactivation [12], an occurrence which can lead to the under estimation of the potential of pathogen exposure to populations as affected organisms will typically not be detected by standard assays [13].

Many bacteria have an innate resistance to ionising and non-ionising radiation. For example, the extremophile bacterium *Deinococcus radiodurans*, can grow continuously in the presence of chronic radiation (6 kilorads/h), and also it can survive acute exposures to gamma radiation exceeding 1,500 kilorads without significant loss of viability [14]. The same organism is also resistant to UV light at fluences of up to 2000 J/m^2^ [15]. *D. radiodurans* is able to tolerate these conditions due to a number of factors such as, having multiple copies of the genome to reduce the chance of a gene mutation being passed to a progeny [14]; high recombination rates [16], [17]; a multilayered protective cell wall [15] and extremely efficient DNA repair systems [14], [16], [18]. For example, following an exposure to 1.75 mrad (the dose required to achieve a survival of 37%, which degrades the cells’ four chromosomes into about 500 fragments), Daly and Minton determined that there were as many as 175 crossovers per chromosome (700 crossovers per nucleoid) undergoing repair [17]. *D. radiodurans* can also effectively repair up to 100 DNA double-strand breaks per chromosome after exposure to a 1.5 mrad dose of gamma radiation [19] and subsequent studies showed that repair is a two-stage *RecA*-dependent process involving the unique, extended synthesis-dependent single-strand DNA annealing (ESDSA) system followed by a more conventional protein-mediated double-strand break repair [20]–[22]. Other domains of life also contain highly UV-resistant organisms. For instance, the eggs of the helminth, *Ascaris suum* were resistant to UV fluences of up to 1000 J/m^2^ [23] and the Tardigrade *Ramazzottius varieornatus* survived UVC fluences of up to 2500 J/m^2^ [24].

We have previously shown that use of a copper-alginate bead is capable of lysing *E. coli* cells up to approximately 10^8^ cells/ml, in simulated wastewater under laboratory conditions [25], and have further enhanced their efficacy through use within the ‘vortex bioreactor’ which creates complex swirl-type flow regimes and effective mixing [26]. As a new technology being developed as an alternative to, among others, UV sterilisation for waste water treatment, this work represents the first direct comparison of vortex bioreactor performance. Here, we focus on *E. coli* and *E. faecalis* as model organisms in water treatment efficacy studies and *D. radiodurans* as an organism with a natural resistance to existing treatment systems. During the following study, we compare the use of UV treatment with the vortex bioreactor, for the sterilisation of simulated waste streams containing increasing colour, humic acid and suspended solid concentrations.

## Methods

### Copper-alginate bead manufacture

Copper-alginate beads were made according to the methodology described by ourselves previously [26]. The beads were stored in sterile millipore water at 4°C prior to use, and allowed to warm to 37°C in an incubator (Thermo scientific, Hemel Hempstead, UK) before use.

### Swirl flow bioreactor design

**Figure 1:**
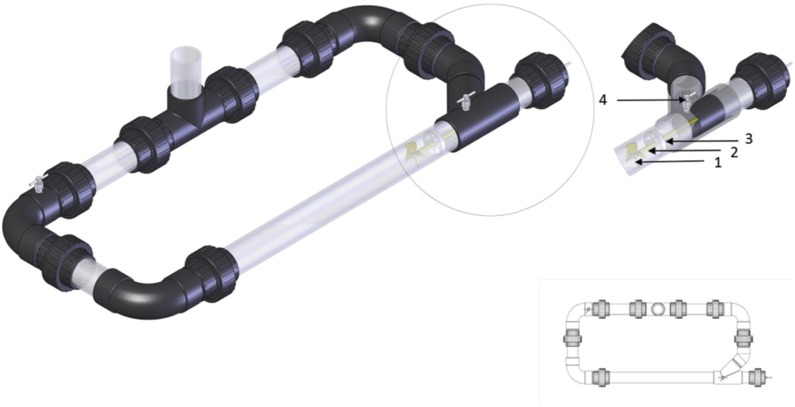
The general configuration of the swirl flow reactor, with the drive system highlighted. 1: Impeller; 2: Drive shaft; 3: Drive shaft stabiliser; 4: Air release valve. Adapted from Thomas *et al* ^26^.

The reactor was manufactured from 60.4 mm outside diameter (OD) clear polycarbonate tube and 60.4 mm internal diameter (ID) acrylonitrile butadiene styrene (ABS) fittings. The system was of a recirculating design with a total length of 1300 mm, a width of 550 mm and a working volume of 6.5 litres. The system consisted of a four blade stainless steel propeller (Fig. 1, item 1 [316 s, 58 mm diameter; pitch 63 mm]) retained on a 7 mm stainless steel shaft (Fig. 1, item 2 [316 s]). The shaft was located via a circular metal support with a 25 mm stainless steel ball bearing at the centre through which the shaft ran (Fig. 1, item 3). The shaft ran centrally through the tube and emerged through a female connector; the fluid integrity was maintained by dual stainless steel cutlass bearings (27.6 mm diameter) housed in a 60 mm diameter butyl bung, through which the shaft ran through centrally. The shaft was attached to a 450 watt variable speed motor (0–2400 rpm [Bosch]). The propeller induced flow in a clockwise direction and swirl formation and flow rates were determined by adjustment propeller velocity. Filling and sampling was facilitated by a vertical inlet via a T-connector with a 150 mm length of polycarbonate tube attached. Air was added to the system via a 400 mm length of 3 mm ID stainless steel tube, sealed at one end, with 0.5 mm holes drilled at 10 mm intervals along the length. Dissolved oxygen (DO) concentration in the test media was monitored via a 240 volt solenoid (GHL profilux, Germany) connected to a Profilux 3 controller (GHL Profilux, Germany), which also controlled temperature via a 300 watt stainless steel tube heater (Hydor, Wiltshire, UK) inserted through the sampling tube [26].

### Growth of Bacteria

*E. coli* ATCC 11775 from −80°C stored stocks was inoculated into 50 ml of sterilised Luria-Bertani (LB) broth and *Enterococcus faecalis* ATCC 19434 was grown in Davies minimal media (Sigma Aldrich, Poole, UK). *Deinococcus radiodurans* ATCC 13939 was grown in TGY broth Bacto tryptone 10 g/l, yeast extract 5 g/l and D-glucose 1 g/l (BD, New Jersey, USA). All cultures were incubated overnight at 37°C in a shaking incubator (Bibby scientific, Staffordshire, UK) at 150 rpm; 1 ml of this stock was then added to 250 ml of media, which was incubated overnight under the same conditions. The bacterial cell numbers were then determined using the Gram staining method and subsequent enumeration using an improved Neubauer hemocytometer.

### Toxicity assay procedure

All experiments were performed in the swirl flow reactor described above, all assays were performed in a media containing 0.1 g/l Bacto peptone and 0.02 g/l Yeast extract (both Sigma Aldrich, Poole, UK). Briefly, cultures of organisms were added at the appropriate concentration in 6.5 litres of assay media (∼10^8^ cells/ml for *E. coli*, ∼10^7^ cells/ml for *E. faecalis* and 10^8^ cells/ml for *D. radiodurans*) and then added directly to the swirl flow reactor. The speed of rotation was set to 956 rpm (+/−20 rpm) and the temperature was maintained at 37°C for 30 minutes to allow acclimation. Alginate beads (650 g) were then added aseptically giving a final copper concentration of 0.25 mg/ml. 1 ml of sample was then removed at 15 minute intervals for microbial enumeration. Suspended solids were added between 0 and 1000 mg/l, as insoluble cellulose. FeCl_2_ and humic acid (all Sigma Aldrich, Poole, UK) were prepared as 100 g/l stock solutions, filter sterilised using a 0.2 µm cellulose filter (Millipore Ltd, Dundee, UK) and stored at 4°C prior to use. Each sample was serial diluted at 4°C in 1/8 strength Ringers solution (Fisher, Leicester, UK) and plated onto 90 mm Petri dishes containing LB, Davies or TGY agar media. The Petri dishes were then incubated overnight at 37°C and colonies counted. All plates were reassessed on a daily basis for a two week period for any potential regrowth. All treatments were prepared in triplicate, and technical triplicate replicates were also performed.

### Formulae

Using a continuous-flow system operated with laminar flow and assuming a constant UV-C intensity inside the quartz sleeve, inactivation of the test bacteria as a function of intensity is assumed to comply with first-order kinetics [27]. This exponential relationship can be approximated by the equation: *N_1_/N_0_ = e^−KIT^,* where *N_1_* is bacterial density after exposure (colony-forming units (CFU) per millilitre of effluent), *N_0_* is initial bacterial density (CFU per millilitre of influent), *N_1_/N_0_* is the bacterial survival ratio, *K* is the inactivation rate constant (square centimetres per microwatt minute, cm^2^/µW min), *I* is the intensity of received UV-C radiation (microwatts per square centimetre), *T* is time of exposure to UV-C radiation (min), and e is equal to 2.7182. Thus *K = (log N_o_ – log N_1_)/IT*. Exposure time (*T*) is calculated from *T = Q/V*, where *T* is time of exposure to UV-C radiation (minutes), *Q* is total capacity of the transparent part of reactor (litres), and *V* is the flow rate of water passing through the reactor (litres per minute). If the bacterial survival ratio *(N_1_/N_0_)* is to be expressed in terms of percentage, then *S = (100)e^−KIT^*, where S is bacterial survival (percent). Fluence *(F)*, or radiation dose, is the product of UV-C intensity *(I)* and exposure time *(T), F = IT.* Thus, fluence was regulated by varying the *V* through the system.

### UV sterilisation system

UV-C flux was measured using a Ultraviolet UVC Light Meter (Sper Scientific, AZ, USA). The UV-C was provided by a low-pressure 14 Watt germicidal lamp (generating 3.7 watt of UV-C), with a quartz sleeve (Bio-logic, Grenoble, France) and the flow was provided by a peristaltic pump (Watson Marlow, Falmouth, UK).

## Results

**Figure 2:**
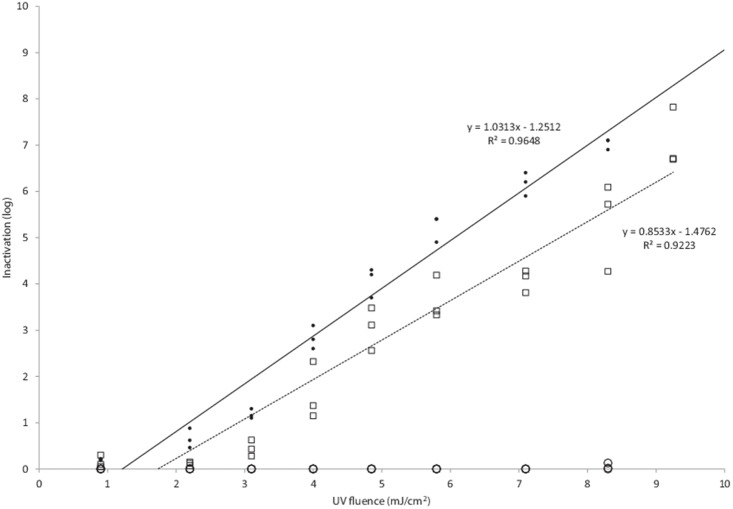
The UV-fluence response curves for *E. coli* ATCC 1775 (•); *Enterococcus faecalis* ATCC 19434 (□) and *Deinococcus radiodurans* ATCC 13939 (○).

Both *E. coli* ATCC 1775 and *Enterococcus faecalis* ATCC 19434 were unaffected by UV fluences of below approximately 1.25 and 1.47 mJ/cm^2^ respectively (Fig. 2), and at a fluence of 4. 96 mJ/cm^2^ a mean log inactivation of 3.86 was observed for *E. coli* ATCC 1775 and 3.16 for *E. faecalis* ATCC 19434. The R^2^ values for the linear response was 0.9648 for *E. coli* ATCC 1775 and 0.9223 for *E. faecalis* ATCC 19434 and the dose response of fluence against log inactivation was linear according to *y* = 1.0313*x*–1.2512 and *y* = 0.8533x–1.4762 respectively. No effect on survival rate was observed for *D. radiodurans* with UV fluences up to 8.16 mJ/cm^2^.

**Figure 3:**
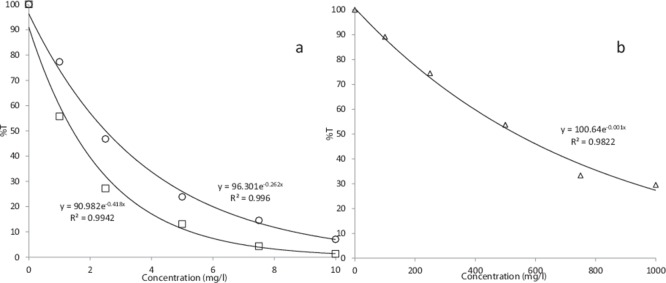
The decrease in transmission (%T) with increasing concentration of FeCl_2_ (□); tannic acid (○) and insoluble cellulose (Δ).

**Figure 4:**
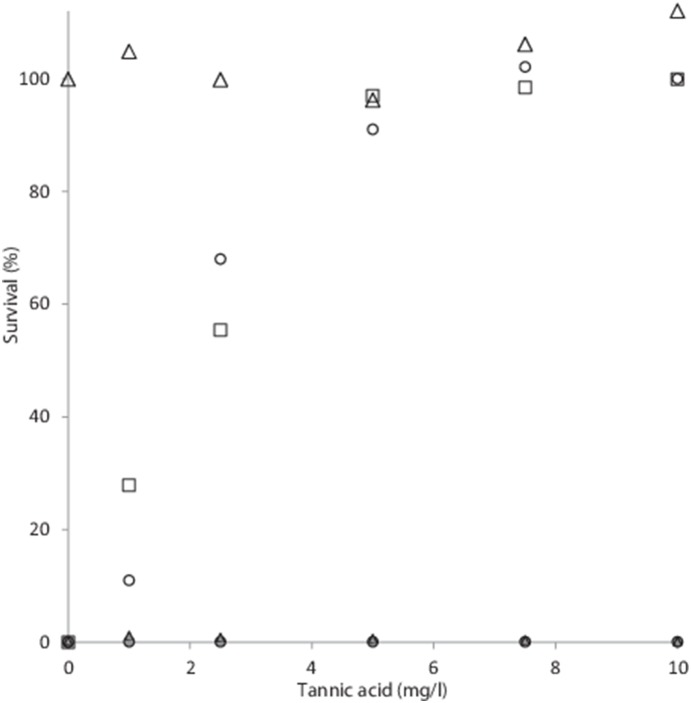
Survival of *E. faecalis* (○), *E. coli* ATCC 1775 (□) and *D. radiodurans* (Δ) under a UV-C fluence of 10 mJ/cm^2^ with increasing concentrations of tannic acid. Grey markers indicate copper-alginate bead treatment.

**Figure 5:**
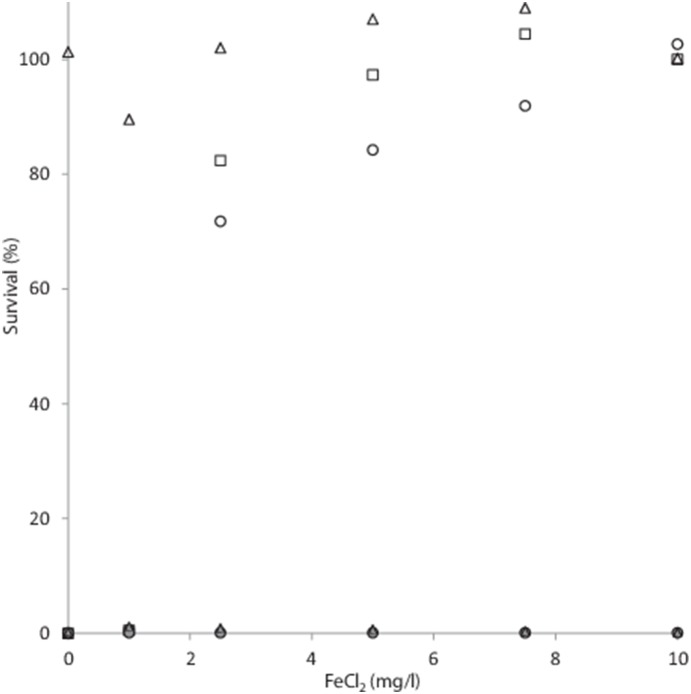
Survival of *E. faecalis* (○), *E. coli* ATCC 1775 (□) and *D. radiodurans* (Δ) under a UV-C fluence of 10 mJ/cm^2^ with increasing concentrations of FeCl_2_. Grey markers indicate copper-alginate bead treatment.

Transmission at 254 nm for increasing doses of FeCl_2_, tannic acid and cellulose decreased with dose, in a natural log (ln) related manner (Fig. 3). For tannic acid the equation was y = 96.301*e* ^−0.262x^ (R^2^ = 0.996); for FeCl_2_ y = 90.982*e* ^−418x^ ((R^2^ = 0.9942) Fig. 3a) and for cellulose, y = 100.64*e* ^−0.001x^ ((R^2^ = 0.9822) Fig. 3b). At a fluence of 10 mJ/cm^2^ the survival of both *E. coli* ATCC 1775 and *E. faecalis* ATCC 19434 increased rapidly with tannic acid concentrations and even a 1 mg/l dose resulted in 11% survival of *E. faecalis* and 27.82% survival for *E. coli* (Fig. 4). No effect was observed on *D. radiodurans* at a fluence of 10 mJ/cm^2^. At concentrations of 5 mg/l tannic acid 91.6% and 97% of each bacterium were viable, respectively (Fig. 4). No antibacterial effects were seen in either bacterium with tannic acid concentrations up to 10 mg/l (Fig. 4). With FeCl_2_ no significant effect on survival was observed with concentrations up to 1 mg/l. However, when the dose increased to 2.5 mg/l, bacterial viability increased rapidly and at 2.5 mg/l 71.79% of *E. faecalis* were viable and 82.35% of *E. coli* (Fig. 5). At a dose of 7.5 mg/l, 92% of *E. faecalis* were viable and *E. coli* even increased in viable cells of 4.47% over starting values (Fig. 5).

**Figure 6:**
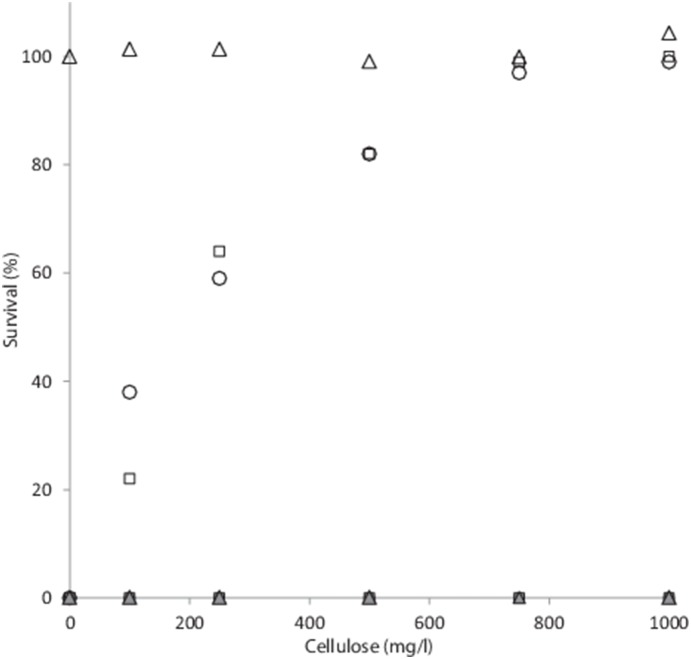
Survival of *E. faecalis* (○), *E. coli* ATCC 1775 (□) and *D. radiodurans* (Δ) under a UV-C fluence of 10 mJ/cm^2^ with increasing concentrations of cellulose. Grey markers indicate copper-alginate bead treatment.

With the copper alginate bead treatment, no effect of increasing tannic acid or FeCl_2_ concentrations was observed, and no survival was observed with either treatments at the concentrations used during the study (Figs. 4 and 5). Additionally, the Cu-alginate bead treatment was unaffected by suspended solid concentration up to the 1000 mg/l tested (Fig. 6); whereas UV-C at a fluence of 10 mJ/cm^2^ showed decreased effectiveness in a (*ln*) linear manner (r^2^ = 0.989), with 38% of *E. faecalis* cells still viable at a suspended solid concentration of 100 mg/l, increasing to 99.97% at the 1000 mg/l concentration (Fig. 6). The survival of *E. coli* cells also positively correlated with suspended solid concentration in a (*ln*) linear manner ((r^2^ = 0.914) Fig. 6), with 22% survival observed at 100 mg/l, 64.3% at 300 mg/l and 99.27% at 750 mg/l (Fig. 6).

## Discussion

The treatment of contaminated water by UV-C is an established and effective technique [6], [29], that is widely utilised in developed countries [2]. The mechanism of action [30], [31] and dose responses [1], [6], [8]–[10], [32] (and Fig. 2) are well characterised for many organisms, and UV-C absorbing chemicals within the waste stream ensure the reliance of upstream treatments to increase efficacy [1], [2]. However, in developing countries, such systems may not be suitable for decontaminating water, due to high capital expenditure, running costs and highly coloured or solid loaded waste streams [4].

Here we describe a comparison of the efficacy of a simple swirl flow based reactor (known as the vortex bioreactor), containing copper-alginate beads with UV-C technology on simulated waste streams containing high concentrations of colour and suspended solids, and established faecal coliform markers in addition to the highly resilient bacteria, *D. radiodurans*. We have previously illustrated the potential of copper-alginate beads as an antimicrobial agent [25] and the use of a swirl flow reactor in combination with the beads for the same purpose [26]. This study illustrates that such a technique is highly effective in reducing the viability of several organisms in conditions that limit the use of UV-C.

Obviously the effectiveness of a UV-C based system is reliant on the transmission of radiation of a suitable wave length through the medium that is treated, and both humic acids and FeCl_2_ are known to effect this parameter [7], [8]. As can be seen in Fig. 3, increasing doses of FeCl_2_, tannic acid and cellulose decreased transmission, in a log (*ln*) related manner. Indeed the expected inverse log (*ln*) response is observed for both *E. faecalis* and *E. coli* with increasing concentrations of tannic acid and FeCl_2_ (Figs. 4 and 5). No effect on the efficacy of the copper-alginate beads on the viability either *E. faecalis* and *E. coli* was observed with any dose of either tannic acid or FeCl_2_ (Figs. 4 and 5), and furthermore, the radiation resistant bacteria *D. radiodurans* showed minimum survival under any test conditions in the vortex bioreactor during this study (Figs. 4, 5 and 6). This result is more surprising; as the bacterium is one of the hardiest known, able to resist acute high doses of both ionising and non-ionising radiation in doses up to 20 times higher than *E. coli* with almost no loss in viability [14], [17], and is known to survive elevated external concentrations of copper due to a copper-responsive gene cluster that encodes *CopA*, which is a copper-transporting P1-type ATPase, *CopZ*, which is a copper metallochaperone, and *CsoR*, which is a copper-sensing repressor [33]. We reported previously that there was a synergistic effect between the copper and alginate, probably due to the spacing and coordinates of the dendritic copper particles within the alginate matrix [25]. This effectively increases the surface area for exposure, as the copper is distributed in a non-random pattern. The susceptibility of *D. radiodurans* to copper exposure within the vortex bioreactor observed during this study is suggestive of an irreversible membrane disruption mechanism of action for the system rather than radical induced damage to intracellular contents (such as DNA) which *D. radiodurans* is capable of withstanding [15].

Elevated suspended solid concentrations present a two-fold problem for the elimination of microorganisms from waste streams. Firstly, as confirmed by Fig. 3b, these present an effective barrier to UV-C transmission [9], [11] and secondly, an effective environment for microbes to live which is totally shielded from UV-C [34]. As shown is Fig. 6, an increase in suspended solids resulted in increased survival for both *E. faecalis* and *E. coli* with the UV treatment. No such increase was observed for the copper-alginate-vortex bioreactor treatment, which suggests that both the dosage of Cu was unaffected by the presence of increasing solids, and that the bacteria adhering or inside the particles were still effectively destroyed. This may be due to the charge on the Cu ion in solution, which at the neutral pH used during this study is predominately as Cu^2+^ [25]. Most suspended solids contain a net negative charge [35], [36] thus resulting in an attraction between these particles and highly bioactive Cu^2+^ ion, a property which enables the use of clay to remove aqueous copper from mine water runoff [37]. This ionic attraction may increase the Cu^2+^ concentration on and inside the suspended solid particles.

We did not have the capacity to test the system extensively on eukaryotic pathogens such as helminthic organisms that are endemic in many third world populations [38] and cause considerable morbidity and financial burden on these populations [39], [40]. Limited trials with *Ascaris suum* eggs revealed that neither the vortex bioreactor nor UV-C exposure conditions used herein had any impact on egg structural integrity (data not shown). However, despite copper alginate beads apparently having limited value in decreasing *A. suum* egg mortality, the possibility of using another agent within the vortex bioreactor remains a possibility and is an active area of research for us.

We have shown during this study that the combination of a swirl flow reactor and an antimicrobial agent (‘vortex bioreactor’), in this case copper alginate beads, is a promising technique for the remediation of contaminated water in waste streams recalcitrant to UV-C treatment. The reactor is easy to construct from commonly available plumbing parts and may prove a versatile and powerful tool in waste water treatment in developing countries. However, several issues still require further work, for example, bead integrity needs to be improved and the antimicrobial spectrum of the agent increased to include viruses and helminths, but the general principle of the system is one, we believe, worth further investigation.
